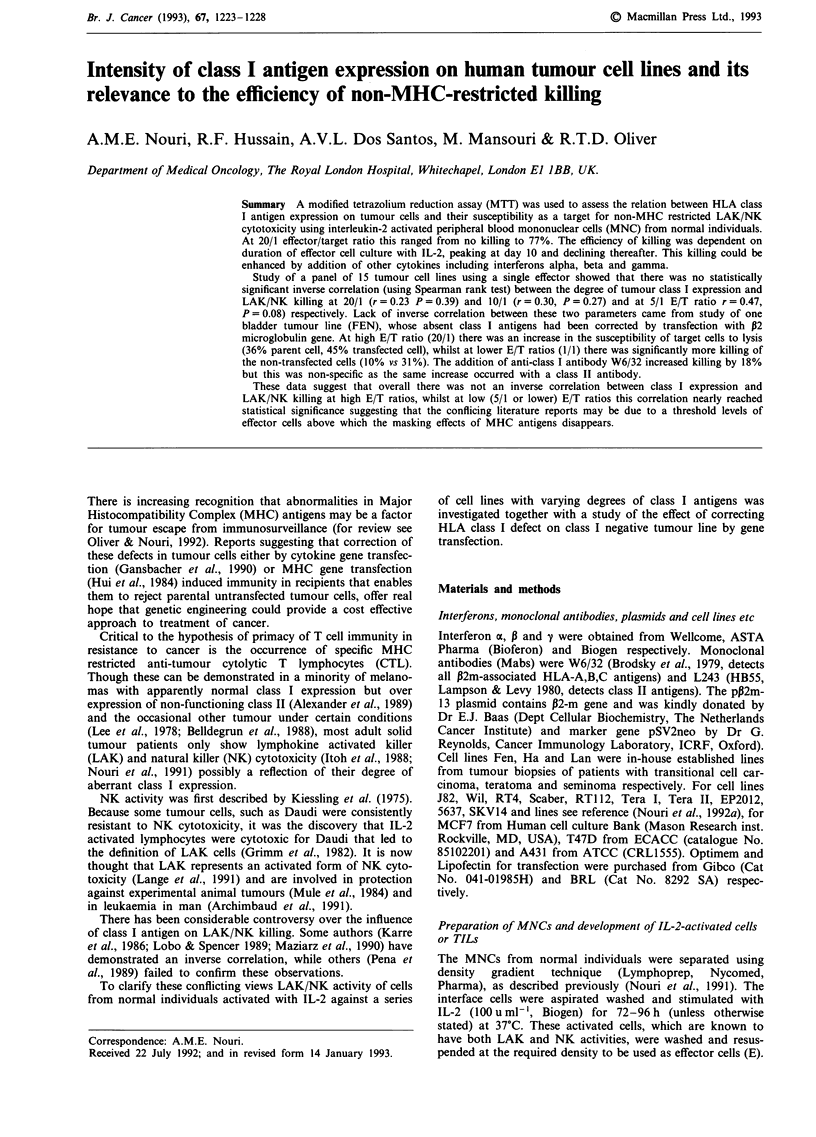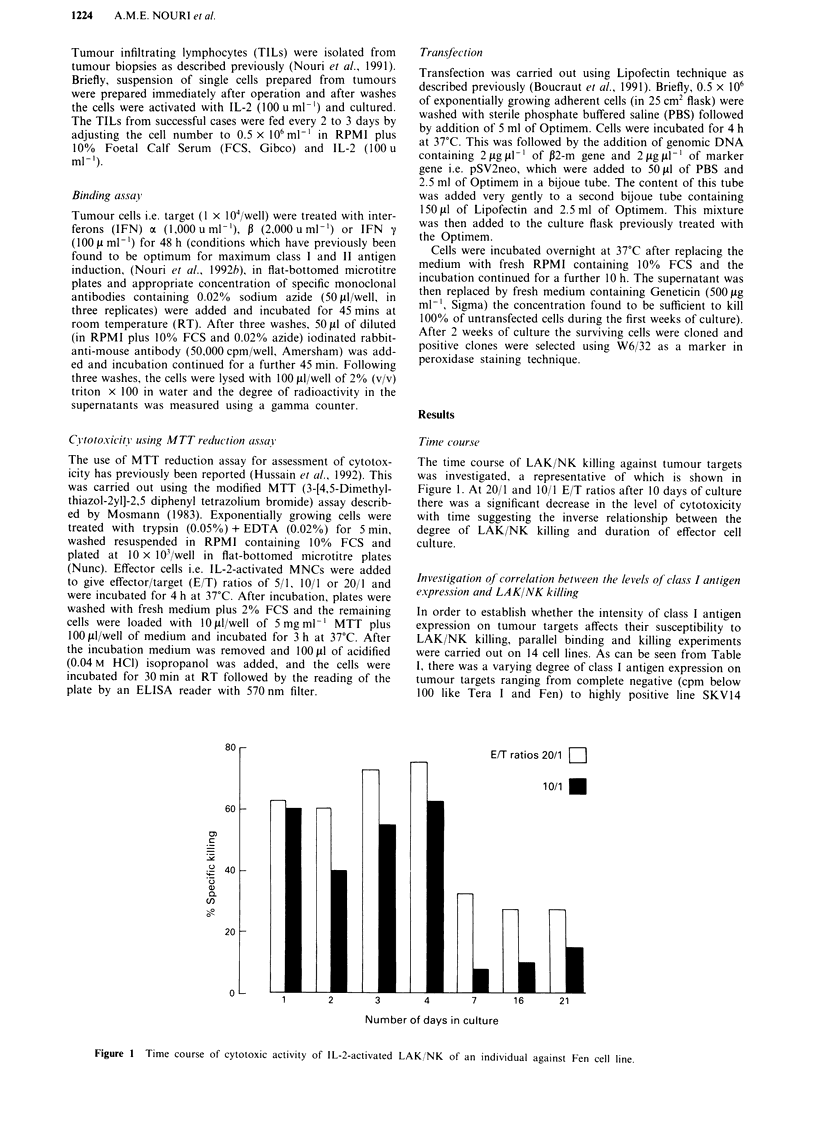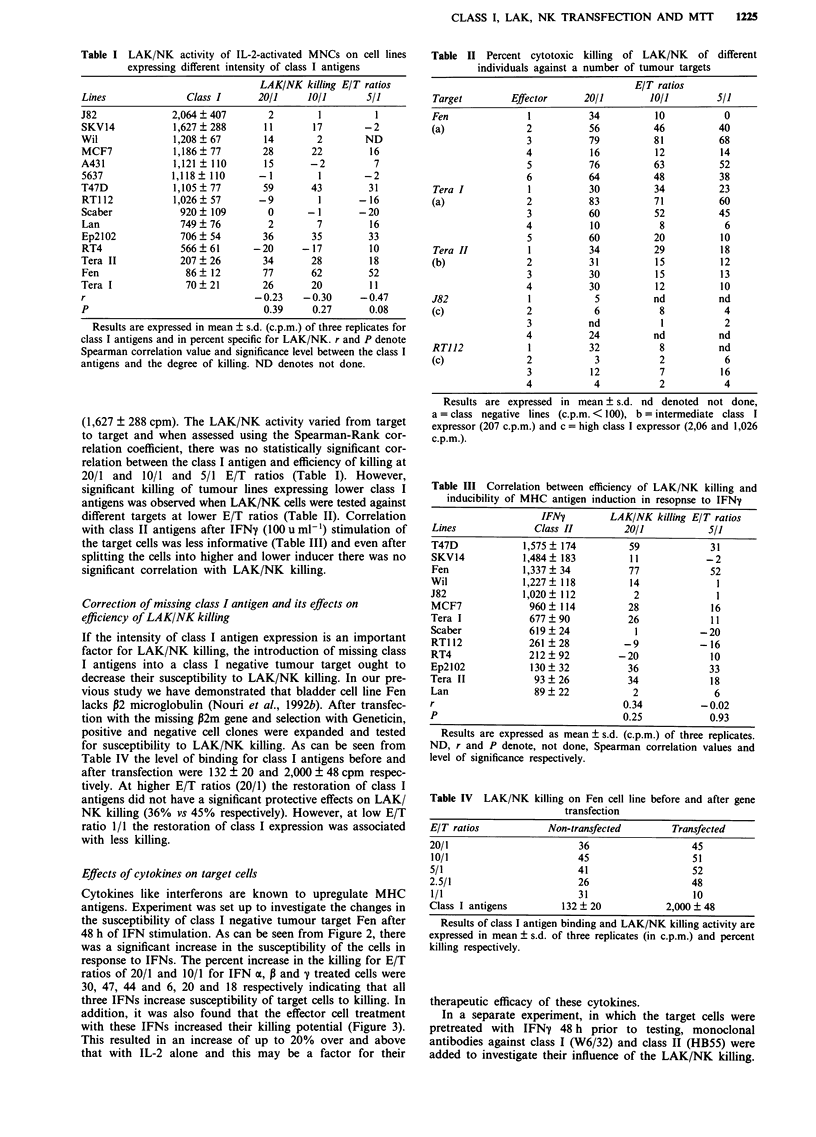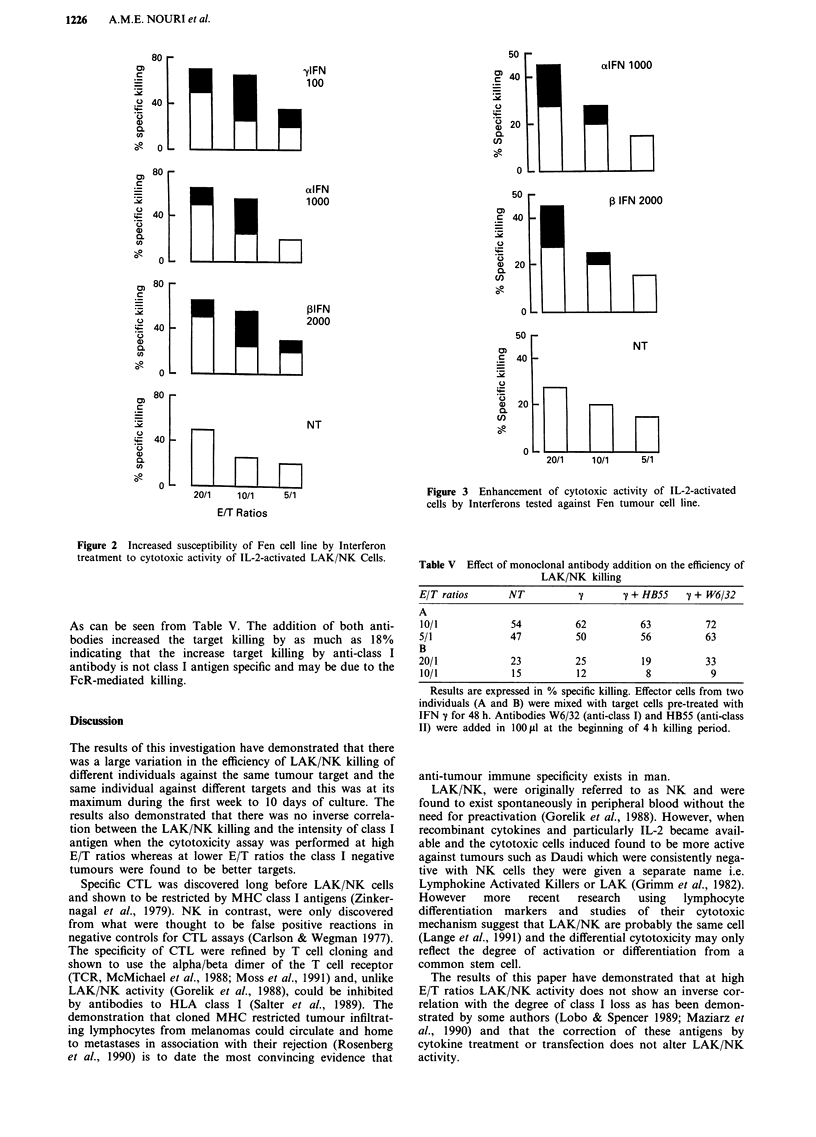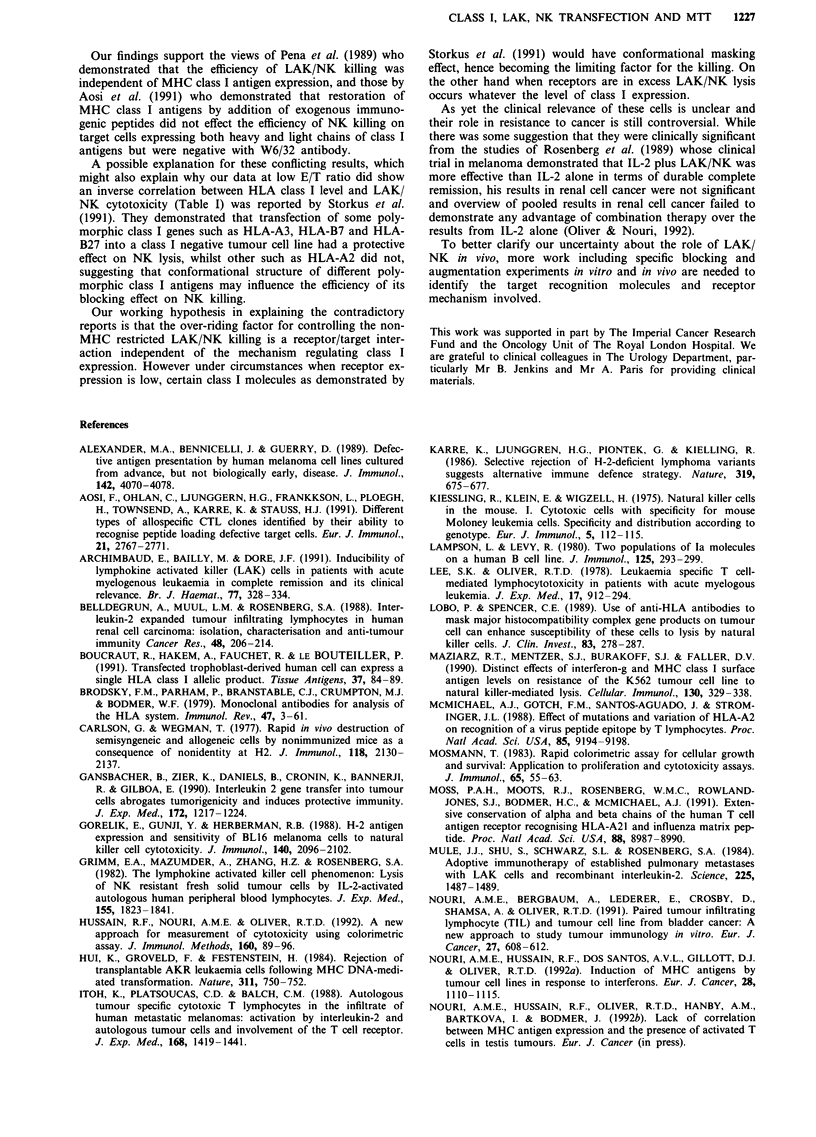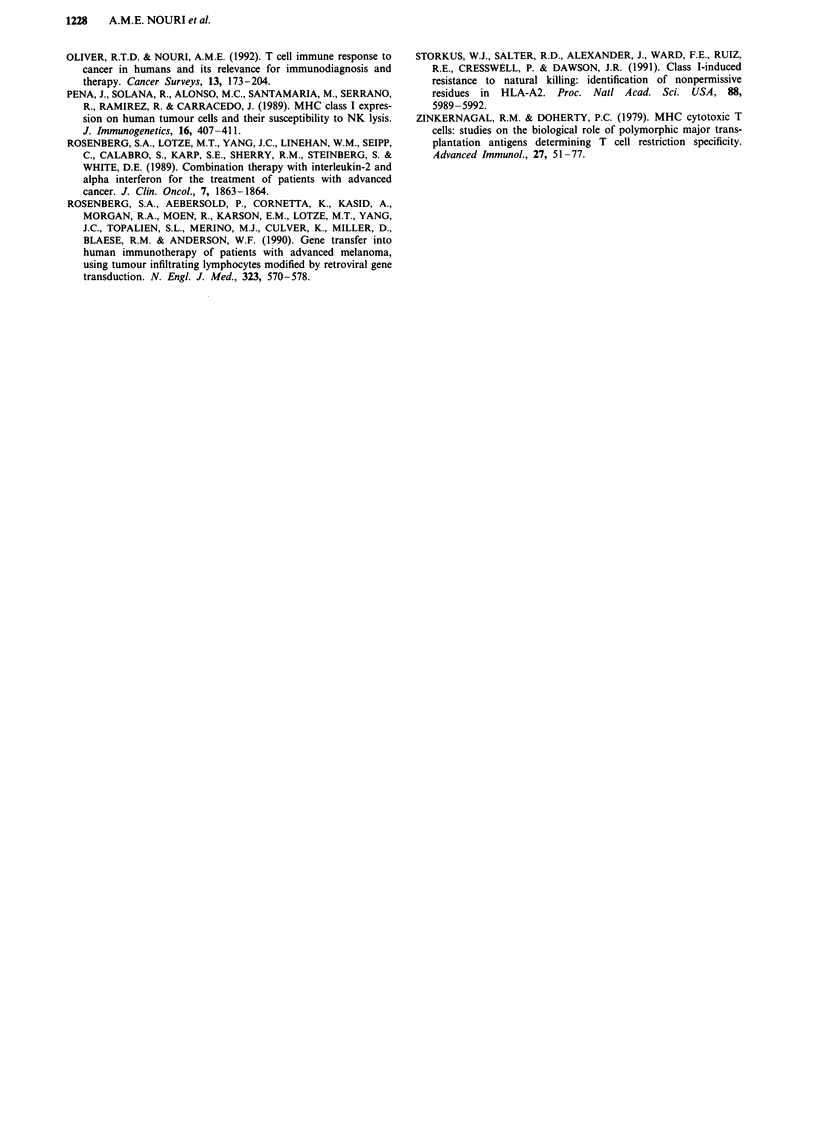# Intensity of class I antigen expression on human tumour cell lines and its relevance to the efficiency of non-MHC-restricted killing.

**DOI:** 10.1038/bjc.1993.229

**Published:** 1993-06

**Authors:** A. M. Nouri, R. F. Hussain, A. V. Dos Santos, M. Mansouri, R. T. Oliver

**Affiliations:** Department of Medical Oncology, Royal London Hospital, Whitechapel, UK.

## Abstract

A modified tetrazolium reduction assay (MTT) was used to assess the relation between HLA class I antigen expression on tumour cells and their susceptibility as a target for non-MHC restricted LAK/NK cytotoxicity using interleukin-2 activated peripheral blood mononuclear cells (MNC) from normal individuals. At 20/1 effector/target ratio this ranged from no killing to 77%. The efficiency of killing was dependent on duration of effector cell culture with IL-2, peaking at day 10 and declining thereafter. This killing could be enhanced by addition of other cytokines including interferons alpha, beta and gamma. Study of a panel of 15 tumour cell lines using a single effector showed that there was no statistically significant inverse correlation (using Spearman rank test) between the degree of tumour class I expression and LAK/NK killing at 20/1 (r = 0.23 P = 0.39) and 10/1 (r = 0.30, P = 0.27) and at 5/1 E/T ratio r = 0.47, P = 0.08) respectively. Lack of inverse correlation between these two parameters came from study of one bladder tumour line (FEN), whose absent class I antigens had been corrected by transfection with beta 2 microglobulin gene. At high E/T ratio (20/1) there was an increase in the susceptibility of target cells to lysis (36% parent cell, 45% transfected cell), whilst at lower E/T ratios (1/1) there was significantly more killing of the non-transfected cells (10% vs 31%). The addition of anti-class I antibody W6/32 increased killing by 18% but this was non-specific as the same increase occurred with a class II antibody. These data suggest that overall there was not an inverse correlation between class I expression and LAK/NK killing at high E/T ratios, whilst at low (5/1 or lower) E/T ratios this correlation nearly reached statistical significance suggesting that the conflicting literature reports may be due to a threshold levels of effector cells above which the masking effects of MHC antigens disappears.


Br. J. Cancer (1993), 67, 1223–1228 © Macmillan Press Ltd., 1993

### Intensity of class I antigen expression on human tumour cell lines and its relevance to the efficiency of non-MHC-restricted killing

A.M.E. Nouri, R.F. Hussain, A.V.L. Dos Santos, M. Mansouri & R.T.D. Oliver

*Department of Medical Oncology, The Royal London Hospital, Whitechapel, London E1 1BB, UK.*

**Summary** A modified tetrazolium reduction assay (MTT) was used to assess the relation between HLA class I antigen expression on tumour cells and their susceptibility as a target for non-MHC restricted LAK/NK cytotoxicity using interleukin-2 activated peripheral blood mononuclear cells (MNC) from normal individuals. At 20/1 effector/target ratio this ranged from no killing to 77%. The efficiency of killing was dependent on duration of effector cell culture with IL-2, peaking at day 10 and declining thereafter. This killing could be enhanced by addition of other cytokines including interferons alpha, beta and gamma.

Study of a panel of 15 tumour cell lines using a single effector showed that there was no statistically significant inverse correlation (using Spearman rank test) between the degree of tumour class I expression and LAK/NK killing at 20/1 ($r = 0.23$ $P = 0.39$) and 10/1 ($r = 0.30$, $P = 0.27$) and at 5/1 E/T ratio $r = 0.47$, $P = 0.08$) respectively. Lack of inverse correlation between these two parameters came from study of one bladder tumour line (FEN), whose absent class I antigens had been corrected by transfection with β2 microglobulin gene. At high E/T ratio (20/1) there was an increase in the susceptibility of target cells to lysis (36% parent cell, 45% transfected cell), whilst at lower E/T ratios (1/1) there was significantly more killing of the non-transfected cells (10% *vs* 31%). The addition of anti-class I antibody W6/32 increased killing by 18% but this was non-specific as the same increase occurred with a class II antibody.

These data suggest that overall there was not an inverse correlation between class I expression and LAK/NK killing at high E/T ratios, whilst at low (5/1 or lower) E/T ratios this correlation nearly reached statistical significance suggesting that the conficing literature reports may be due to a threshold levels of effector cells above which the masking effects of MHC antigens disappears.

There is increasing recognition that abnormalities in Major Histocompatibility Complex (MHC) antigens may be a factor for tumour escape from immunosurveillance (for review see Oliver & Nouri, 1992). Reports suggesting that correction of these defects in tumour cells either by cytokine gene transfection (Gansbacher *et al.*, 1990) or MHC gene transfection (Hui *et al.*, 1984) induced immunity in recipients that enables them to reject parental untransfected tumour cells, offer real hope that genetic engineering could provide a cost effective approach to treatment of cancer.

Critical to the hypothesis of primacy of T cell immunity in resistance to cancer is the occurrence of specific MHC restricted anti-tumour cytolytic T lymphocytes (CTL). Though these can be demonstrated in a minority of melanomas with apparently normal class I expression but over expression of non-functioning class II (Alexander *et al.*, 1989) and the occasional other tumour under certain conditions (Lee *et al.*, 1978; Belldegrun *et al.*, 1988), most adult solid tumour patients only show lymphokine activated killer (LAK) and natural killer (NK) cytotoxicity (Itoh *et al.*, 1988; Nouri *et al.*, 1991) possibly a reflection of their degree of aberrant class I expression.

NK activity was first described by Kiessling *et al.* (1975). Because some tumour cells, such as Daudi were consistently resistant to NK cytotoxicity, it was the discovery that IL-2 activated lymphocytes were cytotoxic for Daudi that led to the definition of LAK cells (Grimm *et al.*, 1982). It is now thought that LAK represents an activated form of NK cytotoxicity (Lange *et al.*, 1991) and are involved in protection against experimental animal tumours (Mule *et al.*, 1984) and in leukaemia in man (Archimbaud *et al.*, 1991).

There has been considerable controversy over the influence of class I antigen on LAK/NK killing. Some authors (Karre *et al.*, 1986; Lobo & Spencer 1989; Maziarz *et al.*, 1990) have demonstrated an inverse correlation, while others (Pena *et al.*, 1989) failed to confirm these observations.

To clarify these conflicting views LAK/NK activity of cells from normal individuals activated with IL-2 against a series of cell lines with varying degrees of class I antigens was investigated together with a study of the effect of correcting HLA class I defect on class I negative tumour line by gene transfection.

Correspondence: A.M.E. Nouri.
Received 22 July 1992; and in revised form 14 January 1993.

### Materials and methods

### *Interferons, monoclonal antibodies, plasmids and cell lines etc*

Interferon α, β and γ were obtained from Wellcome, ASTA Pharma (Bioferon) and Biogen respectively. Monoclonal antibodies (Mabs) were W6/32 (Brodsky *et al.*, 1979, detects all β2m-associated HLA-A,B,C antigens) and L243 (HB55, Lampson & Levy 1980, detects class II antigens). The pβ2m-13 plasmid contains β2-m gene and was kindly donated by Dr E.J. Baas (Dept Cellular Biochemistry, The Netherlands Cancer Institute) and marker gene pSV2neo by Dr G. Reynolds, Cancer Immunology Laboratory, ICRF, Oxford). Cell lines Fen, Ha and Lan were in-house established lines from tumour biopsies of patients with transitional cell carcinoma, teratoma and seminoma respectively. For cell lines J82, Wil, RT4, Scaber, RT112, Tera I, Tera II, EP2012, 5637, SKV14 and lines see reference (Nouri *et al.*, 1992*a*), for MCF7 from Human cell culture Bank (Mason Research inst. Rockville, MD, USA), T47D from ECACC (catalogue No. 85102201) and A431 from ATCC (CRL1555). Optimem and Lipofectin for transfection were purchased from Gibco (Cat No. 041-01985H) and BRL (Cat No. 8292 SA) respectively.

### *Preparation of MNCs and development of IL-2-activated cells or TILs*

The MNCs from normal individuals were separated using density gradient technique (Lymphoprep, Nycomed, Pharma), as described previously (Nouri *et al.*, 1991). The interface cells were aspirated washed and stimulated with IL-2 ($100\,\mathrm{u\,ml^{-1}}$, Biogen) for 72–96 h (unless otherwise stated) at 37°C. These activated cells, which are known to have both LAK and NK activities, were washed and resuspended at the required density to be used as effector cells (E).

Tumour infiltrating lymphocytes (TILs) were isolated from tumour biopsies as described previously (Nouri et al., 1991). Briefly, suspension of single cells prepared from tumours were prepared immediately after operation and after washes the cells were activated with IL-2 (100 u $ml^{-1}$) and cultured. The TILs from successful cases were fed every 2 to 3 days by adjusting the cell number to $0.5 \times 10^6 ml^{-1}$ in RPMI plus 10% Foetal Calf Serum (FCS, Gibco) and IL-2 (100 u $ml^{-1}$).

*Binding assay*

Tumour cells i.e. target ($1 \times 10^4$/well) were treated with interferons (IFN) $\alpha$ (1,000 u $ml^{-1}$), $\beta$ (2,000 u $ml^{-1}$) or IFN $\gamma$ (100 $\mu$ $ml^{-1}$) for 48 h (conditions which have previously been found to be optimum for maximum class I and II antigen induction, (Nouri et al., 1992b), in flat-bottomed microtitre plates and appropriate concentration of specific monoclonal antibodies containing 0.02% sodium azide (50 µl/well, in three replicates) were added and incubated for 45 mins at room temperature (RT). After three washes, 50 µl of diluted (in RPMI plus 10% FCS and 0.02% azide) iodinated rabbit-anti-mouse antibody (50,000 cpm/well, Amersham) was added and incubation continued for a further 45 min. Following three washes, the cells were lysed with 100 µl/well of 2% (v/v) triton × 100 in water and the degree of radioactivity in the supernatants was measured using a gamma counter.

*Cytotoxicity using MTT reduction assay*

The use of MTT reduction assay for assessment of cytotoxicity has previously been reported (Hussain et al., 1992). This was carried out using the modified MTT (3-[4,5-Dimethylthiazol-2yl]-2,5 diphenyl tetrazolium bromide) assay described by Mosmann (1983). Exponentially growing cells were treated with trypsin (0.05%) + EDTA (0.02%) for 5 min, washed resuspended in RPMI containing 10% FCS and plated at $10 \times 10^3$/well in flat-bottomed microtitre plates (Nunc). Effector cells i.e. IL-2-activated MNCs were added to give effector/target (E/T) ratios of 5/1, 10/1 or 20/1 and were incubated for 4 h at 37°C. After incubation, plates were washed with fresh medium plus 2% FCS and the remaining cells were loaded with 10 µl/well of 5 mg $ml^{-1}$ MTT plus 100 µl/well of medium and incubated for 3 h at 37°C. After the incubation medium was removed and 100 µl of acidified (0.04 M HCl) isopropanol was added, and the cells were incubated for 30 min at RT followed by the reading of the plate by an ELISA reader with 570 nm filter.

*Transfection*

Transfection was carried out using Lipofectin technique as described previously (Boucraut et al., 1991). Briefly, $0.5 \times 10^6$ of exponentially growing adherent cells (in 25 $cm^2$ flask) were washed with sterile phosphate buffered saline (PBS) followed by addition of 5 ml of Optimem. Cells were incubated for 4 h at 37°C. This was followed by the addition of genomic DNA containing 2 $\mu g \mu l^{-1}$ of $\beta$2-m gene and 2 $\mu g \mu l^{-1}$ of marker gene i.e. pSV2neo, which were added to 50 µl of PBS and 2.5 ml of Optimem in a bijoue tube. The content of this tube was added very gently to a second bijoue tube containing 150 µl of Lipofectin and 2.5 ml of Optimem. This mixture was then added to the culture flask previously treated with the Optimem.

Cells were incubated overnight at 37°C after replacing the medium with fresh RPMI containing 10% FCS and the incubation continued for a further 10 h. The supernatant was then replaced by fresh medium containing Geneticin (500 µg $ml^{-1}$, Sigma) the concentration found to be sufficient to kill 100% of untransfected cells during the first weeks of culture). After 2 weeks of culture the surviving cells were cloned and positive clones were selected using W6/32 as a marker in peroxidase staining technique.

### Results

*Time course*

The time course of LAK/NK killing against tumour targets was investigated, a representative of which is shown in Figure 1. At 20/1 and 10/1 E/T ratios after 10 days of culture there was a significant decrease in the level of cytotoxicity with time suggesting the inverse relationship between the degree of LAK/NK killing and duration of effector cell culture.

*Investigation of correlation between the levels of class I antigen expression and LAK/NK killing*

In order to establish whether the intensity of class I antigen expression on tumour targets affects their susceptibility to LAK/NK killing, parallel binding and killing experiments were carried out on 14 cell lines. As can be seen from Table I, there was a varying degree of class I antigen expression on tumour targets ranging from complete negative (cpm below 100 like Tera I and Fen) to highly positive line SKV14

**Figure 1** Time course of cytotoxic activity of IL-2-activated LAK/NK of an individual against Fen cell line.

**Table I** LAK/NK activity of IL-2-activated MNCs on cell lines expressing different intensity of class I antigens

| | | *LAK/NK killing E/T ratios* | | |
|---|---|---|---|---|
| *Lines* | *Class I* | *20/1* | *10/1* | *5/1* |
| J82 | 2,064 ± 407 | 2 | 1 | 1 |
| SKV14 | 1,627 ± 288 | 11 | 17 | −2 |
| Wil | 1,208 ± 67 | 14 | 2 | ND |
| MCF7 | 1,186 ± 77 | 28 | 22 | 16 |
| A431 | 1,121 ± 110 | 15 | −2 | 7 |
| 5637 | 1,118 ± 110 | −1 | 1 | −2 |
| T47D | 1,105 ± 77 | 59 | 43 | 31 |
| RT112 | 1,026 ± 57 | −9 | 1 | −16 |
| Scaber | 920 ± 109 | 0 | −1 | −20 |
| Lan | 749 ± 76 | 2 | 7 | 16 |
| Ep2102 | 706 ± 54 | 36 | 35 | 33 |
| RT4 | 566 ± 61 | −20 | −17 | 10 |
| Tera II | 207 ± 26 | 34 | 28 | 18 |
| Fen | 86 ± 12 | 77 | 62 | 52 |
| Tera I | 70 ± 21 | 26 | 20 | 11 |
| *r* | | −0.23 | −0.30 | −0.47 |
| *P* | | 0.39 | 0.27 | 0.08 |

Results are expressed in mean ± s.d. (c.p.m.) of three replicates for class I antigens and in percent specific for LAK/NK. *r* and *P* denote Spearman correlation value and significance level between the class I antigens and the degree of killing. ND denotes not done.

(1,627 ± 288 cpm). The LAK/NK activity varied from target to target and when assessed using the Spearman-Rank correlation coefficient, there was no statistically significant correlation between the class I antigen and efficiency of killing at 20/1 and 10/1 and 5/1 E/T ratios (Table I). However, significant killing of tumour lines expressing lower class I antigens was observed when LAK/NK cells were tested against different targets at lower E/T ratios (Table II). Correlation with class II antigens after IFNγ (100 u ml$^{-1}$) stimulation of the target cells was less informative (Table III) and even after splitting the cells into higher and lower inducer there was no significant correlation with LAK/NK killing.

### *Correction of missing class I antigen and its effects on efficiency of LAK/NK killing*

If the intensity of class I antigen expression is an important factor for LAK/NK killing, the introduction of missing class I antigens into a class I negative tumour target ought to decrease their susceptibility to LAK/NK killing. In our previous study we have demonstrated that bladder cell line Fen lacks β2 microglobulin (Nouri *et al.*, 1992*b*). After transfection with the missing β2m gene and selection with Geneticin, positive and negative cell clones were expanded and tested for susceptibility to LAK/NK killing. As can be seen from Table IV the level of binding for class I antigens before and after transfection were 132 ± 20 and 2,000 ± 48 cpm respectively. At higher E/T ratios (20/1) the restoration of class I antigens did not have a significant protective effects on LAK/NK killing (36% *vs* 45% respectively). However, at low E/T ratio 1/1 the restoration of class I expression was associated with less killing.

### *Effects of cytokines on target cells*

Cytokines like interferons are known to upregulate MHC antigens. Experiment was set up to investigate the changes in the susceptibility of class I negative tumour target Fen after 48 h of IFN stimulation. As can be seen from Figure 2, there was a significant increase in the susceptibility of the cells in response to IFNs. The percent increase in the killing for E/T ratios of 20/1 and 10/1 for IFN α, β and γ treated cells were 30, 47, 44 and 6, 20 and 18 respectively indicating that all three IFNs increase susceptibility of target cells to killing. In addition, it was also found that the effector cell treatment with these IFNs increased their killing potential (Figure 3). This resulted in an increase of up to 20% over and above that with IL-2 alone and this may be a factor for their therapeutic efficacy of these cytokines.

**Table II** Percent cytotoxic killing of LAK/NK of different individuals against a number of tumour targets

| | | *E/T ratios* | | |
|---|---|---|---|---|
| *Target* | *Effector* | *20/1* | *10/1* | *5/1* |
| *Fen* | 1 | 34 | 10 | 0 |
| (a) | 2 | 56 | 46 | 40 |
| | 3 | 79 | 81 | 68 |
| | 4 | 16 | 12 | 14 |
| | 5 | 76 | 63 | 52 |
| | 6 | 64 | 48 | 38 |
| *Tera I* | 1 | 30 | 34 | 23 |
| (a) | 2 | 83 | 71 | 60 |
| | 3 | 60 | 52 | 45 |
| | 4 | 10 | 8 | 6 |
| | 5 | 60 | 20 | 10 |
| *Tera II* | 1 | 34 | 29 | 18 |
| (b) | 2 | 31 | 15 | 12 |
| | 3 | 30 | 15 | 13 |
| | 4 | 30 | 12 | 10 |
| *J82* | 1 | 5 | nd | nd |
| (c) | 2 | 6 | 8 | 4 |
| | 3 | nd | 1 | 2 |
| | 4 | 24 | nd | nd |
| *RT112* | 1 | 32 | 8 | nd |
| (c) | 2 | 3 | 2 | 6 |
| | 3 | 12 | 7 | 16 |
| | 4 | 4 | 2 | 4 |

Results are expressed in mean ± s.d. nd denoted not done, a = class negative lines (c.p.m. < 100), b = intermediate class I expressor (207 c.p.m.) and c = high class I expressor (2,06 and 1,026 c.p.m.).

**Table III** Correlation between efficiency of LAK/NK killing and inducibility of MHC antigen induction in resopnse to IFNγ

| | *IFNγ* | *LAK/NK killing E/T ratios* | |
|---|---|---|---|
| *Lines* | *Class II* | *20/1* | *5/1* |
| T47D | 1,575 ± 174 | 59 | 31 |
| SKV14 | 1,484 ± 183 | 11 | −2 |
| Fen | 1,337 ± 34 | 77 | 52 |
| Wil | 1,227 ± 118 | 14 | 1 |
| J82 | 1,020 ± 112 | 2 | 1 |
| MCF7 | 960 ± 114 | 28 | 16 |
| Tera I | 677 ± 90 | 26 | 11 |
| Scaber | 619 ± 24 | 1 | −20 |
| RT112 | 261 ± 28 | −9 | −16 |
| RT4 | 212 ± 92 | −20 | 10 |
| Ep2102 | 130 ± 32 | 36 | 33 |
| Tera II | 93 ± 26 | 34 | 18 |
| Lan | 89 ± 22 | 2 | 6 |
| *r* | | 0.34 | −0.02 |
| *P* | | 0.25 | 0.93 |

Results are expressed as mean ± s.d. (c.p.m.) of three replicates. ND, *r* and *P* denote, not done, Spearman correlation values and level of significance respectively.

**Table IV** LAK/NK killing on Fen cell line before and after gene transfection

| *E/T ratios* | *Non-transfected* | *Transfected* |
|---|---|---|
| 20/1 | 36 | 45 |
| 10/1 | 45 | 51 |
| 5/1 | 41 | 52 |
| 2.5/1 | 26 | 48 |
| 1/1 | 31 | 10 |
| Class I antigens | 132 ± 20 | 2,000 ± 48 |

Results of class I antigen binding and LAK/NK killing activity are expressed in mean ± s.d. of three replicates (in c.p.m.) and percent killing respectively.

In a separate experiment, in which the target cells were pretreated with IFNγ 48 h prior to testing, monoclonal antibodies against class I (W6/32) and class II (HB55) were added to investigate their influence of the LAK/NK killing.

**Figure 2** Increased susceptibility of Fen cell line by Interferon treatment to cytotoxic activity of IL-2-activated LAK/NK Cells.

**Figure 3** Enhancement of cytotoxic activity of IL-2-activated cells by Interferons tested against Fen tumour cell line.

**Table V** Effect of monoclonal antibody addition on the efficiency of LAK/NK killing

| *E/T ratios* | *NT* | *γ* | *γ + HB55* | *γ + W6/32* |
|---|---|---|---|---|
| A | | | | |
| 10/1 | 54 | 62 | 63 | 72 |
| 5/1 | 47 | 50 | 56 | 63 |
| B | | | | |
| 20/1 | 23 | 25 | 19 | 33 |
| 10/1 | 15 | 12 | 8 | 9 |

Results are expressed in % specific killing. Effector cells from two individuals (A and B) were mixed with target cells pre-treated with IFN γ for 48 h. Antibodies W6/32 (anti-class I) and HB55 (anti-class II) were added in 100 μl at the beginning of 4 h killing period.

As can be seen from Table V. The addition of both antibodies increased the target killing by as much as 18% indicating that the increase target killing by anti-class I antibody is not class I antigen specific and may be due to the FcR-mediated killing.

### Discussion

The results of this investigation have demonstrated that there was a large variation in the efficiency of LAK/NK killing of different individuals against the same tumour target and the same individual against different targets and this was at its maximum during the first week to 10 days of culture. The results also demonstrated that there was no inverse correlation between the LAK/NK killing and the intensity of class I antigen when the cytotoxicity assay was performed at high E/T ratios whereas at lower E/T ratios the class I negative tumours were found to be better targets.

Specific CTL was discovered long before LAK/NK cells and shown to be restricted by MHC class I antigens (Zinkernagal *et al*., 1979). NK in contrast, were only discovered from what were thought to be false positive reactions in negative controls for CTL assays (Carlson & Wegman 1977). The specificity of CTL were refined by T cell cloning and shown to use the alpha/beta dimer of the T cell receptor (TCR, McMichael *et al*., 1988; Moss *et al*., 1991) and, unlike LAK/NK activity (Gorelik *et al*., 1988), could be inhibited by antibodies to HLA class I (Salter *et al*., 1989). The demonstration that cloned MHC restricted tumour infiltrating lymphocytes from melanomas could circulate and home to metastases in association with their rejection (Rosenberg *et al*., 1990) is to date the most convincing evidence that anti-tumour immune specificity exists in man.

LAK/NK, were originally referred to as NK and were found to exist spontaneously in peripheral blood without the need for preactivation (Gorelik *et al*., 1988). However, when recombinant cytokines and particularly IL-2 became available and the cytotoxic cells induced found to be more active against tumours such as Daudi which were consistently negative with NK cells they were given a separate name i.e. Lymphokine Activated Killers or LAK (Grimm *et al*., 1982). However more recent research using lymphocyte differentiation markers and studies of their cytotoxic mechanism suggest that LAK/NK are probably the same cell (Lange *et al*., 1991) and the differential cytotoxicity may only reflect the degree of activation or differentiation from a common stem cell.

The results of this paper have demonstrated that at high E/T ratios LAK/NK activity does not show an inverse correlation with the degree of class I loss as has been demonstrated by some authors (Lobo & Spencer 1989; Maziarz *et al*., 1990) and that the correction of these antigens by cytokine treatment or transfection does not alter LAK/NK activity.

Our findings support the views of Pena *et al.* (1989) who demonstrated that the efficiency of LAK/NK killing was independent of MHC class I antigen expression, and those by Aosi *et al.* (1991) who demonstrated that restoration of MHC class I antigens by addition of exogenous immunogenic peptides did not effect the efficiency of NK killing on target cells expressing both heavy and light chains of class I antigens but were negative with W6/32 antibody.

A possible explanation for these conflicting results, which might also explain why our data at low E/T ratio did show an inverse correlation between HLA class I level and LAK/NK cytotoxicity (Table I) was reported by Storkus *et al.* (1991). They demonstrated that transfection of some polymorphic class I genes such as HLA-A3, HLA-B7 and HLA-B27 into a class I negative tumour cell line had a protective effect on NK lysis, whilst other such as HLA-A2 did not, suggesting that conformational structure of different polymorphic class I antigens may influence the efficiency of its blocking effect on NK killing.

Our working hypothesis in explaining the contradictory reports is that the over-riding factor for controlling the non-MHC restricted LAK/NK killing is a receptor/target interaction independent of the mechanism regulating class I expression. However under circumstances when receptor expression is low, certain class I molecules as demonstrated by Storkus *et al.* (1991) would have conformational masking effect, hence becoming the limiting factor for the killing. On the other hand when receptors are in excess LAK/NK lysis occurs whatever the level of class I expression.

As yet the clinical relevance of these cells is unclear and their role in resistance to cancer is still controversial. While there was some suggestion that they were clinically significant from the studies of Rosenberg *et al.* (1989) whose clinical trial in melanoma demonstrated that IL-2 plus LAK/NK was more effective than IL-2 alone in terms of durable complete remission, his results in renal cell cancer were not significant and overview of pooled results in renal cell cancer failed to demonstrate any advantage of combination therapy over the results from IL-2 alone (Oliver & Nouri, 1992).

To better clarify our uncertainty about the role of LAK/NK *in vivo*, more work including specific blocking and augmentation experiments *in vitro* and *in vivo* are needed to identify the target recognition molecules and receptor mechanism involved.

This work was supported in part by The Imperial Cancer Research Fund and the Oncology Unit of The Royal London Hospital. We are grateful to clinical colleagues in The Urology Department, particularly Mr B. Jenkins and Mr A. Paris for providing clinical materials.

### References

ALEXANDER, M.A., BENNICELLI, J. & GUERRY, D. (1989). Defective antigen presentation by human melanoma cell lines cultured from advance, but not biologically early, disease. *J. Immunol.*, **142**, 4070–4078.

AOSI, F., OHLAN, C., LJUNGGERN, H.G., FRANKKSON, L., PLOEGH, H., TOWNSEND, A., KARRE, K. & STAUSS, H.J. (1991). Different types of allospecific CTL clones identified by their ability to recognise peptide loading defective target cells. *Eur. J. Immunol.*, **21**, 2767–2771.

ARCHIMBAUD, E., BAILLY, M. & DORE, J.F. (1991). Inducibility of lymphokine activated killer (LAK) cells in patients with acute myelogenous leukaemia in complete remission and its clinical relevance. *Br. J. Haemat.*, **77**, 328–334.

BELLDEGRUN, A., MUUL, L.M. & ROSENBERG, S.A. (1988). Interleukin-2 expanded tumour infiltrating lymphocytes in human renal cell carcinoma: isolation, characterisation and anti-tumour immunity *Cancer Res.*, **48**, 206–214.

BOUCRAUT, R., HAKEM, A., FAUCHET, R. & LE BOUTEILLER, P. (1991). Transfected trophoblast-derived human cell can express a single HLA class I allelic product. *Tissue Antigens*, **37**, 84–89.

BRODSKY, F.M., PARHAM, P., BRANSTABLE, C.J., CRUMPTON, M.J. & BODMER, W.F. (1979). Monoclonal antibodies for analysis of the HLA system. *Immunol. Rev.*, **47**, 3–61.

CARLSON, G. & WEGMAN, T. (1977). Rapid *in vivo* destruction of semisyngeneic and allogeneic cells by nonimmunized mice as a consequence of nonidentity at H2. *J. Immunol.*, **118**, 2130–2137.

GANSBACHER, B., ZIER, K., DANIELS, B., CRONIN, K., BANNERJI, R. & GILBOA, E. (1990). Interleukin 2 gene transfer into tumour cells abrogates tumorigenicity and induces protective immunity. *J. Exp. Med.*, **172**, 1217–1224.

GORELIK, E., GUNJI, Y. & HERBERMAN, R.B. (1988). H-2 antigen expression and sensitivity of BL16 melanoma cells to natural killer cell cytotoxicity. *J. Immunol.*, **140**, 2096–2102.

GRIMM, E.A., MAZUMDER, A., ZHANG, H.Z. & ROSENBERG, S.A. (1982). The lymphokine activated killer cell phenomenon: Lysis of NK resistant fresh solid tumour cells by IL-2-activated autologous human peripheral blood lymphocytes. *J. Exp. Med.*, **155**, 1823–1841.

HUSSAIN, R.F., NOURI, A.M.E. & OLIVER, R.T.D. (1992). A new approach for measurement of cytotoxicity using colorimetric assay. *J. Immunol. Methods*, **160**, 89–96.

HUI, K., GROVELD, F. & FESTENSTEIN, H. (1984). Rejection of transplantable AKR leukaemia cells following MHC DNA-mediated transformation. *Nature*, **311**, 750–752.

ITOH, K., PLATSOUCAS, C.D. & BALCH, C.M. (1988). Autologous tumour specific cytotoxic T lymphocytes in the infiltrate of human metastatic melanomas: activation by interleukin-2 and autologous tumour cells and involvement of the T cell receptor. *J. Exp. Med.*, **168**, 1419–1441.

KARRE, K., LJUNGGREN, H.G., PIONTEK, G. & KIELLING, R. (1986). Selective rejection of H-2-deficient lymphoma variants suggests alternative immune defence strategy. *Nature*, **319**, 675–677.

KIESSLING, R., KLEIN, E. & WIGZELL, H. (1975). Natural killer cells in the mouse. I. Cytotoxic cells with specificity for mouse Moloney leukemia cells. Specificity and distribution according to genotype. *Eur. J. Immunol.*, **5**, 112–115.

LAMPSON, L. & LEVY, R. (1980). Two populations of Ia molecules on a human B cell line. *J. Immunol.*, **125**, 293–299.

LEE, S.K. & OLIVER, R.T.D. (1978). Leukaemia specific T cell-mediated lymphocytotoxicity in patients with acute myelogous leukemia. *J. Exp. Med.*, **17**, 912–294.

LOBO, P. & SPENCER, C.E. (1989). Use of anti-HLA antibodies to mask major histocompatibility complex gene products on tumour cell can enhance susceptibility of these cells to lysis by natural killer cells. *J. Clin. Invest.*, **83**, 278–287.

MAZIARZ, R.T., MENTZER, S.J., BURAKOFF, S.J. & FALLER, D.V. (1990). Distinct effects of interferon-g and MHC class I surface antigen levels on resistance of the K562 tumour cell line to natural killer-mediated lysis. *Cellular. Immunol.*, **130**, 329–338.

MCMICHAEL, A.J., GOTCH, F.M., SANTOS-AGUADO, J. & STROMINGER, J.L. (1988). Effect of mutations and variation of HLA-A2 on recognition of a virus peptide epitope by T lymphocytes. *Proc. Natl Acad. Sci. USA*, **85**, 9194–9198.

MOSMANN, T. (1983). Rapid colorimetric assay for cellular growth and survival: Application to proliferation and cytotoxicity assays. *J. Immunol.*, **65**, 55–63.

MOSS, P.A.H., MOOTS, R.J., ROSENBERG, W.M.C., ROWLAND-JONES, S.J., BODMER, H.C., & MCMICHAEL, A.J. (1991). Extensive conservation of alpha and beta chains of the human T cell antigen receptor recognising HLA-A21 and influenza matrix peptide. *Proc. Natl Acad. Sci. USA*, **88**, 8987–8990.

MULE, J.J., SHU, S., SCHWARZ, S.L. & ROSENBERG, S.A. (1984). Adoptive immunotherapy of established pulmonary metastases with LAK cells and recombinant interleukin-2. *Science*, **225**, 1487–1489.

NOURI, A.M.E., BERGBAUM, A., LEDERER, E., CROSBY, D., SHAMSA, A. & OLIVER, R.T.D. (1991). Paired tumour infiltrating lymphocyte (TIL) and tumour cell line from bladder cancer: A new approach to study tumour immunology *in vitro*. *Eur. J. Cancer*, **27**, 608–612.

NOURI, A.M.E., HUSSAIN, R.F., DOS SANTOS, A.V.L., GILLOTT, D.J. & OLIVER, R.T.D. (1992*a*). Induction of MHC antigens by tumour cell lines in response to interferons. *Eur. J. Cancer*, **28**, 1110–1115.

NOURI, A.M.E., HUSSAIN, R.F., OLIVER, R.T.D., HANBY, A.M., BARTKOVA, I. & BODMER, J. (1992*b*). Lack of correlation between MHC antigen expression and the presence of activated T cells in testis tumours. *Eur. J. Cancer* (in press).

OLIVER, R.T.D. & NOURI, A.M.E. (1992). T cell immune response to cancer in humans and its relevance for immunodiagnosis and therapy. *Cancer Surveys*, **13**, 173–204.

PENA, J., SOLANA, R., ALONSO, M.C., SANTAMARIA, M., SERRANO, R., RAMIREZ, R. & CARRACEDO, J. (1989). MHC class I expression on human tumour cells and their susceptibility to NK lysis. *J. Immunogenetics*, **16**, 407–411.

ROSENBERG, S.A., LOTZE, M.T., YANG, J.C., LINEHAN, W.M., SEIPP, C., CALABRO, S., KARP, S.E., SHERRY, R.M., STEINBERG, S. & WHITE, D.E. (1989). Combination therapy with interleukin-2 and alpha interferon for the treatment of patients with advanced cancer. *J. Clin. Oncol.*, **7**, 1863–1864.

ROSENBERG, S.A., AEBERSOLD, P., CORNETTA, K., KASID, A., MORGAN, R.A., MOEN, R., KARSON, E.M., LOTZE, M.T., YANG, J.C., TOPALIEN, S.L., MERINO, M.J., CULVER, K., MILLER, D., BLAESE, R.M. & ANDERSON, W.F. (1990). Gene transfer into human immunotherapy of patients with advanced melanoma, using tumour infiltrating lymphocytes modified by retroviral gene transduction. *N. Engl. J. Med.*, **323**, 570–578.

STORKUS, W.J., SALTER, R.D., ALEXANDER, J., WARD, F.E., RUIZ, R.E., CRESSWELL, P. & DAWSON, J.R. (1991). Class I-induced resistance to natural killing: identification of nonpermissive residues in HLA-A2. *Proc. Natl Acad. Sci. USA*, **88**, 5989–5992.

ZINKERNAGAL, R.M. & DOHERTY, P.C. (1979). MHC cytotoxic T cells: studies on the biological role of polymorphic major transplantation antigens determining T cell restriction specificity. *Advanced Immunol.*, **27**, 51–77.